# Neighborhood physical activity facilities predict risk of incident mixed and vascular dementia: The Cardiovascular Health Cognition Study

**DOI:** 10.1002/alz.14387

**Published:** 2024-11-19

**Authors:** Kyle D. Moored, Michael R. Desjardins, Breanna M. Crane, Patrick T. Donahue, Emily A. Richards, Jana A. Hirsch, Gina S. Lovasi, Andrea L. Rosso, Parveen K. Garg, Timothy M. Shields, Frank C. Curriero, Michelle C. Odden, Oscar L. Lopez, Mary L. Biggs, Anne B. Newman, Michelle C. Carlson

**Affiliations:** ^1^ Department of Mental Health Johns Hopkins Bloomberg School of Public Health Baltimore Maryland USA; ^2^ Department of Epidemiology and Spatial Science for Public Health Center Johns Hopkins Bloomberg School of Public Health Baltimore Maryland USA; ^3^ Department of Epidemiology and Biostatistics Drexel University Dornsife School of Public Health Philadelphia Pennsylvania USA; ^4^ Department of Epidemiology University of Pittsburgh School of Public Health Pittsburgh Pennsylvania USA; ^5^ Division of Cardiology University of Southern California Keck School of Medicine Los Angeles California USA; ^6^ Department of Epidemiology and Population Health Stanford University Stanford California USA; ^7^ Departments of Neurology and Psychiatry University of Pittsburgh Pittsburgh Pennsylvania USA; ^8^ Department of Biostatistics University of Washington Seattle Washington USA

**Keywords:** aging, Alzheimer's disease, built environment and health, Geographic Information Systems, urban planning

## Abstract

**INTRODUCTION:**

Neighborhood environments may promote neurocognitive health in part by providing amenities that encourage physical activity. We examined associations between quantity of walkable facilities, including specifically physical activity facilities (e.g., gyms, recreation centers), with risk of incident dementia.

**METHODS:**

Participants included 2923 adults ≥ 65 years old from the Cardiovascular Health Cognition Study (1992–1999), with clinically adjudicated dementia classified over a median 6.0 years of follow‐up. Walkable facilities were measured within 1 km (Euclidean) of home. Self‐reported baseline physical activity was considered a moderator.

**RESULTS:**

In adjusted Cox models, participants with ≥ 2 (vs. 0) physical activity facilities had reduced risk of mixed/vascular dementia, but not Alzheimer's disease, particularly after excluding individuals in the bottom 20th percentile of physical activity (hazard ratio = 0.56, 95% confidence interval: 0.35–0.89).

**DISCUSSION:**

Neighborhood amenities that encourage physical activity may mitigate dementia risk via improved vascular health, especially for individuals with sufficient baseline mobility to use these resources.

**Highlights:**

- We examined associations between nearby walkable facilities and incident dementia.
- Facilities within 1 km were counted via the National Establishment Time Series Database.
- More physical activity facilities predicted lower risk of mixed/vascular dementia.
- No associations were found between walkable facilities and incident Alzheimer's disease.

## 1 BACKGROUND

Alzheimer's disease and related dementias (ADRD) are characterized by severe cognitive impairments that lead to a loss of functional independence in later life.^1^ Lifestyle factors, including physical activity, have been identified as promising non‐pharmacologic preventions for ADRD.^2^, ^3^, ^4^ Importantly, research linking lifestyle factors to ADRD risk has primarily focused on the individual and ignored the environmental context that may contribute to disparities in health and health behaviors.^5^, ^6^

There is growing evidence that neighborhood‐level built and social environments may contribute to cognitive aging. Much research to date has established links between aggregated indices of neighborhood socioeconomic status (nSES) and higher individual‐level cognitive performance.^7^, ^8^, ^9^, ^10^ For example, individuals living in neighborhoods with a higher Area Deprivation Index (ADI; i.e., lower nSES) were shown to have greater dementia incidence, cognitive declines, and cortical thinning in ADRD‐related regions (e.g., middle temporal and entorhinal cortices).^11^, ^12^ Yet, evidence is mixed on whether nSES uniquely predicts dementia risk beyond individual socioeconomic status (SES). A study of racially diverse older adults found that nSES partially mediated the relationship between early life SES and later life cognitive performance.^13^ In contrast, nSES did not predict dementia risk in a study of midlife adults after accounting for their lifespan individual SES, which was a stronger predictor especially among Black individuals.^14^ Notably, these studies did not explore whether nSES uniquely predicted specific dementia subtypes (e.g., Alzheimer's disease [AD], vascular), which may provide further insight on whether these neighborhood resources may act through specific pathologic pathways.

Fewer studies have examined the role of neighborhood walkability on ADRD risk.^15^ Neighborhood walkability refers to built environmental features that promote walking.^16^ These features include residential density, street and transit infrastructure, and destination accessibility. Destination accessibility further encompasses multiple types of walkable resources, such as facilities that promote physical activity (e.g., recreation centers, gyms, parks).^17^, ^18^ Investigating specific walkable resources could pinpoint downstream mechanisms through which nSES may influence cognitive aging and may therefore inform actionable structural interventions to mitigate cognitive risk (e.g., provision of facilities encouraging healthy activity).

Prior studies have found that greater availability and accessibility of neighborhood facilities may promote cognitive functioning in older populations, but that results may differ by subgroup or type of facility.^15^, ^17^, ^19^, ^20^, ^21^ For example, Clarke et al. found that neighborhood institutional resources (e.g., churches, libraries, community centers) were associated cross‐sectionally with higher cognitive performance for White, but not Black, older adults.^19^ Luo et al. further observed that specifically outdoor exercise facilities, but not other community facilities (e.g., volunteer organizations), were related to cognitive performance among Chinese older adults.^22^ Yet, existing studies have primarily examined performance on select cognitive tests, rather than dementia diagnoses from rigorous clinical assessment, and have used cross‐sectional designs or limited follow‐up to examine cognitive change. These limitations make it difficult to establish neighborhood facilities as an upstream protective factor against clinical cognitive impairments.

To address these gaps, we assessed whether objectively measured nSES and quantity of walkable facilities were associated with risk of incident dementia, which was clinically adjudicated for up to 8 years of follow‐up in the Cardiovascular Health Cognition Study (CHCS).^23^, ^24^ Cause‐specific dementia diagnoses (AD, vascular, mixed, other) were adjudicated, allowing us to further examine whether neighborhood measures may act through specific pathological pathways. We hypothesized that nSES would be associated with lower risk of all dementia subtypes. We then investigated associations with quantity of walkable businesses (e.g., retail, food/restaurant, services, religious/educational institutions, etc.) and whether these associations were independent of nSES.^18^ Living near a greater number of walkable facilities, especially those promoting physical activity (e.g., fitness/recreation centers, multiuse facilities [YMCAs]), was previously associated with reduced incident cardiovascular disease in this cohort.^25^ We therefore hypothesized that having a greater number of nearby physical activity facilities would be more strongly associated with reduced risk of mixed/vascular dementia in particular, suggesting an underlying cardiovascular mechanism.^2^, ^3^, ^4^, ^25^

## 2 METHODS

### 2.1 Study sample

Participants were from the CHCS ancillary to the main Cardiovascular Health Study (CHS).^23^, ^24^ CHS was conducted in two primarily urban (Pittsburgh, Pennsylvania and Sacramento, California) and two primarily suburban/rural (Hagerstown, Maryland and Winston‐Salem, North Carolina) study sites. Individuals enrolled in CHS were ≥ 65 years, non‐institutionalized, expected to remain in the area for the next 3 years, and did not require a proxy respondent at baseline.^23^, ^24^ The original CHS cohort consisted of 5201 participants recruited in 1989 and 1990. CHS aimed to include equitable representation of women and men across age groups (65–69, 70–74, 75–79, ≥ 80).To enhance racial diversity, a supplemental cohort comprised of predominantly Black/African‐American individuals (*n* = 687) was enrolled in 1992 and 1993. To be eligible for the ancillary CHCS, participants needed to complete cranial magnetic resonance imaging (MRI) and a Modified Mini Mental State (3MS) exam between 1992 and 1994. All participants provided informed consent and study protocols were approved by the institutional review boards at the University of Washington Coordinating Center and each individual study site.

Of the 3608 individuals enrolled in CHCS, 6 were excluded due to lack of sufficient data for dementia adjudication.^24^ Further, neighborhood measures were only available for individuals with a valid residential address who were deceased at the time of geocoding (August 12, 2016) to mitigate confidentiality concerns from linking potentially identifiable personal and geospatial information.^25^ This resulted in 393 participants being excluded for missing neighborhood measures. An additional 59 participants missing covariate measures were also excluded. Last, 227 prevalent dementia cases were excluded for analyses of incident dementia (see below), resulting in a final analytic sample of 2923 participants.

**RESEARCH IN CONTEXT**

1. **Systematic review**: The authors searched online sources (e.g., PubMed) for related literature using terms such as “neighborhood,” “ walkability,” “physical activity facilities,” “dementia,” and “Alzheimer's disease.”
2. **Interpretation**: A greater number of neighborhood physical activity facilities (e.g., gyms, recreation centers, YMCAs) was associated with reduced risk of incident mixed or vascular dementia, especially for individuals with sufficient current mobility to potentially use these facilities. No associations were found with Alzheimer's disease without vascular pathology, suggesting that these neighborhood resources may operate via a vascular mechanism.
3. **Future directions**: Studies should evaluate the cumulative impact of neighborhood facilities on dementia risk as these data continue to be collected in ongoing cohorts. Additional types of neighborhood facilities that promote cognitive enrichment (e.g., libraries, arts/performance) should be further examined.

### 2.2 Neighborhood characteristics

Neighborhood characteristics were measured as part of the Retail Environment and Cardiovascular Disease (RECVD) project.^26^ Neighborhood exposures were assessed within 1 km radial Euclidean buffers surrounding the geocoded address for each participant at study entry. One km radial buffers have been previously used to quantify environmental exposures within walking distance from the home.^27^, ^28^ In sensitivity analyses, we further examined 5 ‐km radial buffers to better capture exposures within driving distance from home, particularly relevant in more suburban/rural environments.

#### 2.2.1 Neighborhood socioeconomic status

A nSES index was generated using four variables from the 1990 decennial Census at the tract level from the Longitudinal Tract Database:^29^ (1) median household income, (2) median home value, (3) percentage of residents with at least a 4‐year college degree, and (4) percentage of residents with a professional/managerial occupation.^7^, ^30^, ^31^ Buffer‐level measures were derived using area‐proportional weighting of corresponding tract‐level measures. They were then standardized using sample‐specific *z* scores and summed to produce the nSES index (higher = greater socioeconomic resources).^7^, ^30^

#### 2.2.2 Walkable facilities

The National Establishment Time Series (NETS)^32^ database was used to quantify the total number of active businesses in 1990 within each spatial buffer. Facilities were categorized by type through screening standard industrial classification (SIC) codes and supplemental word/chain name searches following a standard protocol.^18^ Our primary exposures included two previously validated measures: (1) all walkable facilities (e.g., retail, restaurants, services, entertainment, religious/educational institutions, etc.), and (2) physical activity facilities (e.g., gym, recreation center, multiuse facility [YMCA]) within the 1 km buffer from the home address.^18^, ^25^ The former measure was intended to capture a broad array of businesses promoting quality of life for older adults.^18^ In contrast, the physical activity facility subcategory included businesses that primarily encouraged sustained physical exercise (light, moderate, or vigorous). All neighborhood measures were assessed using tertiles given high positive skew. Tertiles were approximated for physical activity facilities given the high frequency of 0 facilities (0 [45%], 1 [25%], ≥ 2 [30%]).

### 2.3 Dementia classification

The study outcomes included all‐cause and cause‐specific dementia cases.^23^, ^24^ Prevalent and incident cases were adjudicated in 1998 and 1999 using data collected across 10 annual clinic visits (1989–1999). Data sources included a detailed neuropsychological battery, neurological exam, annual cognitive/behavioral measures, medical records, physician questionnaires, and proxy interviews.^23^ A clinical committee representing each study site classified each dementia case by subtype.^24^ Dementia subtype was classified after review of the MRI using established diagnostic criteria, including the Diagnostic and Statistical Manual of Mental Disorders, Fourth Edition (DSM‐IV),^33^ the National Institute of Neurological and Communicative Disorders and Stroke–Alzheimer's Disease and Related Disorders Association (NINCDS‐ADRDA; AD subtype),^34^ the National Institute of Neurological Diseases and Stroke–Association Internationale pour la Recherche et l'Enseignement en Neurosciences (NINCDS‐AIREN, vascular subtype),^35^ and State of California Alzheimer's Disease Diagnostic and Treatment Centers (ADDTC, vascular subtype).^36^ Generally, cases demonstrated progressive or static cognitive impairments that were severe enough to impact daily functioning and had deficits in at least two cognitive domains.^24^

Time to incident dementia was calculated as number of years from the baseline MRI (1992–1994) to the earliest of: onset of dementia, death, or end of dementia follow‐up (June 1999).^24^, ^27^ Adjudication resulted in 227 prevalent (i.e., at or before baseline MRI) and 464 incident all‐cause dementia cases in the analytic sample. For incident cases, 236 were attributed to AD pathology only, 59 to vascular pathology only, and 148 to mixed AD/vascular pathology, and 21 to other pathologies (e.g., Lewy body). Mixed and vascular cases were combined for cause‐specific analyses, as done previously due to the low number of purely vascular cases.^37^ The median follow‐up time for incident cases was 6.0 years (interquartile range [IQR]: 4.6,6.5).

### 2.4 Covariates

Individual‐level demographic and socioeconomic confounders were included as covariates. Age, sex (male, female), and race were self‐reported at baseline. Analyses were limited to Black and White participants given the proportion of other racial groups was < 1%. Participants self‐reported their education, marital status (married, widowed, divorced/separated, never married), current income (< $12k, [$12–25k), [$25–35k), ≥ $35k, missing/refused), and primary lifetime occupation (professional/technical/managerial/administrative, sales/clerical service, craftsman/machine operator/laborer, farming/forestry, housewife, other, refused to answer). Residential mobility was further assessed as any change in address (yes/no) across the baseline CHS visits before incident dementia started to be assessed (1989–1993). Urbanicity was defined using tertiles of population density within the 1 km buffers (low: 21–2333 people, middle: 2337–6150 people, high: 6150–18,983 people).

We examined baseline physical activity as a potential moderator, given individuals with low activity may be less able to access or use walkable facilities. Physical activity was measured as weekly energy expenditure (kcal/week) from self‐reported household/leisure activities on the Minnesota Leisure Time Questionnaire.^38^ We also explored race as a moderator, as done previously in CHS.^7^ This was done to account for race‐related differences in cognitive testing related to SES and cultural background, as well as differences in SES and social mobility due to systemic racism.^14^

### 2.5 Statistical analysis

Descriptive statistics were examined for all measures for the overall sample and stratified by nSES tertile. To assess differences between nSES tertiles, we used analyses of variance with post hoc pairwise comparisons for continuous variables and chi‐square tests for categorical variables.

We plotted non‐parametric cumulative incidence functions for each neighborhood measure and dementia outcome (all‐cause, AD only, mixed/vascular). We modeled time to dementia using cause‐specific Cox proportional hazards models that were sequentially adjusted for baseline covariates.^39^ Model 1 was age adjusted. Model 2 was further adjusted for demographics (sex, race, marital status, and study site), population density within 1 km (tertiles), and any change in baseline address (yes/no). Model 3 was also adjusted for individual socioeconomic characteristics (education, income, and lifetime occupation). For analyses of walkable facility measures, Model 4 was further adjusted for nSES. Moderation by low baseline physical activity (Model 5) was assessed by excluding individuals meeting the frailty phenotype definition of low activity (bottom 20th percentile, ≤ 225 kcal/week) as done previously.^25^, ^40^ Model 6 was further stratified by race (Black, White), in which tertiles for neighborhood measures were recalculated within racial groups. Plots of Schoenfeld residuals revealed violations of the proportional hazards assumption for study site and occupation, so we also adjusted for their interactions with follow‐up time. Variance estimates for all models were clustered on baseline Census tract to account for the multilevel data structure. Analyses were performed in Stata 18.^41^

## 3 RESULTS

### 3.1 Participant characteristics

The current sample was 75 years old (range: 64–98) on average at study entry, 58% were women, and 86% identified as White (Table 1). Most were married (68%) or widowed (24%), 75% had at least a high school education or General Educational Development credential, and 38% had a professional occupation. Most participants (89%) were residentially stable between 1989 and 1993. The average number of walkable facilities within 1 km of the home was notably high and varied widely across participants (median [IQR]: 38 [12, 87], range: 0–734). Yet, the average number of physical activity facilities was much smaller (median [IQR]: 1 [0, 2], range: 0–10).

**TABLE 1:** Participant characteristics for the overall sample and stratified by neighborhood socioeconomic status tertile (*N* = 2923).

			Neighborhood socioeconomic status tertile (nSES)			
	Overall		Low (−5.71, −1.57)	Moderate (−1.56, 1.26)	High (1.27, 25.8)	
Variable	M ± SD or *N* (%)	Range	M ± SD or *N* (%)	M ± SD or *N* (%)	M ± SD or *N* (%)	*p* value
Age (years)	75.0 ± 4.9	64–98	74.6 ± 5.0	75.1 ± 4.9	75.3 ± 4.9	0.016
Women (vs. men)	1683 (58%)		595 (61%)	548 (56%)	540 (55%)	0.027
Black/African American (vs. White)	414 (14%)		296 (30%)	71 (7%)	47 (5%)	<0.001
CHS site						<0.001
*Winston‐Salem, NC*	703 (24%)		245 (25%)	234 (24%)	224 (23%)	
*Sacramento, CA*	770 (26%)		160 (16%)	239 (25%)	371 (38%)	
*Hagerstown, MD*	677 (23%)		299 (31%)	345 (35%)	33 (3%)	
*Pittsburgh, PA*	773 (26%)		271 (28%)	156 (16%)	346 (36%)	
Marital status						<0.001
*Married*	1975 (68%)		592 (61%)	681 (70%)	702 (72%)	
*Widowed*	698 (24%)		277 (28%)	225 (23%)	196 (20%)	
*Divorced/separated*	134 (5%)		64 (7%)	36 (4%)	34 (3%)	
*Never married*	116 (4%)		42 (4%)	32 (3%)	42 (4%)	
Education						<0.001
* < High school*	716 (25%)		367 (38%)	247 (25%)	102 (10%)	
*High school/GED*	840 (29%)		300 (31%)	293 (30%)	247 (25%)	
* > High school*	1367 (47%)		308 (32%)	434 (45%)	625 (64%)	
Lifetime occupation						<0.001
*Professional*	1102 (38%)		274 (28%)	356 (37%)	472 (48%)	
*Service*	430 (15%)		127 (13%)	147 (15%)	156 (16%)	
*Laborer*	472 (16%)		237 (24%)	153 (16%)	82 (8%)	
*Housewife*	632 (22%)		214 (22%)	220 (23%)	198 (20%)	
*Other*	287 (10%)		123 (13%)	98 (10%)	66 (7%)	
Walkable facilities (1 km)	69.2 ± 82.5	0–734	59.5 ± 75.7	52.2 ± 72.7	95.9 ± 91.2	<0.001
*Physical activity facilities (1 km)*	1.2 ± 1.5	0–10	0.8 ± 1.3	0.9 ± 1.2	1.8 ± 1.8	<0.001

*Note*: *p* values are from analyses of variance (continuous covariates) or chi‐square tests (categorical covariates) of differences by neighborhood socioeconomic status (nSES) tertile.

Abbreviations: CHS, Cardiovascular Health Study; GED, General Educational Development degree; SD, standard deviation.

All covariates differed significantly by nSES tertile (Table 1), except for residential mobility (changing address between 1989 and 1993, *p* > 0.05). Individuals living within the lowest nSES tertile were more likely to be younger, women, and identify as Black or African American. Participants in the lowest nSES tertile tended to have lower numbers of total walkable facilities and a lower count of physical activity facilities within 1 km of home. These differences appeared greatest between the highest tertile and the lower two tertiles, and post hoc pairwise comparisons between the lowest two tertiles were not significant for either measure (*p* > 0.05).

### 3.2 Neighborhood socioeconomic status and incident dementia

Being in the highest (versus lowest) nSES tertile was associated with a 28% reduced hazard of all‐cause dementia (hazard ratio [HR] = 0.72, 95% confidence interval [CI]: 0.56, 0.92), adjusting for age, sex, race, study site, residential mobility, and urbanicity (Table 2, Model 2). This association was attenuated and no longer significant after further adjusting for individual education, income, and lifetime occupation (Table 2, Model 3, HR = 0.81, 95% CI: 0.63, 1.04).

**TABLE 2:** Adjusted associations between neighborhood socioeconomic status and incident all‐cause and cause‐specific dementia (*N* = 2923).

	Model 1^a^		Model 2^b^		Model 3^c^	
	HR (95% CI)	*p* value	HR (95% CI)	*p* value	HR (95% CI)	*p* value
**All‐cause dementia**						
nSES (Ref: Low [−5.7, −1.6])						
Middle (–1.6, 1.3)	0.73 (0.57, 0.92)	0.009	0.79 (0.61, 1.01)	0.059	0.84 (0.66, 1.07)	0.167
High (1.3, 25.8)	0.60 (0.48, 0.75)	<0.001	0.72 (0.56, 0.92)	0.010	0.81 (0.63, 1.04)	0.100
**Alzheimer's disease only subtype**						
nSES (Ref: Low [–5.7, ‐1.6])						
Middle (–1.6, 1.3)	0.74 (0.55, 1.00)	0.047	0.87 (0.62, 1.20)	0.384	0.94 (0.67, 1.31)	0.700
High (1.3, 25.8)	0.60 (0.45, 0.81)	0.001	0.80 (0.56, 1.14)	0.210	0.92 (0.63, 1.33)	0.640
**Mixed/vascular subtype**						
nSES (Ref: Low [–5.7, ‐1.6])						
Middle (–1.6, 1.3)	0.67 (0.47, 0.96)	0.027	0.66 (0.46, 0.94)	0.020	0.69 (0.49, 0.98)	0.036
High (1.3, 25.8)	0.55 (0.40, 0.78)	0.001	0.58 (0.41, 0.82)	0.002	0.63 (0.44, 0.90)	0.011

*Note*: Estimates are hazard ratios comparing risk of incident dementia in the higher neighborhood socioeconomic status tertiles to the lowest tertile.

Abbreviations: CI, confidence interval; HR, hazard ratio, nSES, neighborhood socioeconomic status index.

^a^ Model 1 was age adjusted.

^b^ Model 2 further adjusted for sex (man, woman), race (Black/African American, White), marital status (married, widowed, divorced/separated, never married), study site (Hagerstown, MD; Pittsburgh, PA; Sacramento, CA; Winston‐Salem, NC), residential mobility (any change in address from 1989–1993, yes/no), and urbanicity (tertiles of population density within 1 km).

^c^ Model 3 further adjusted for individual education (< high school, high school degree/General Educational Development degree, > high school), income (< $12k, [$12–25k), [$25–35k), ≥ $35k, missing), and lifetime occupation (professional, service, laborer, housewife, other).

For cause‐specific dementia, being in the highest (versus lowest) nSES tertile was associated with reduced hazard of incident AD (Table 2, HR = 0.60, 95% CI: 0.45, 0.81) and mixed/vascular subtypes (HR = 0.55, 95% CI: 0.40, 0.78) in age‐adjusted models. In fully adjusted models, being in the highest (versus lowest) tertile remained associated with a 37% reduced hazard of mixed/vascular subtype (HR = 0.63, 95% CI: 0.44, 0.90), but was no longer associated with AD subtype (HR = 0.92, 95% CI: 0.63, 1.33).

### 3.3 Neighborhood facilities and incident dementia

**FIGURE 1:**
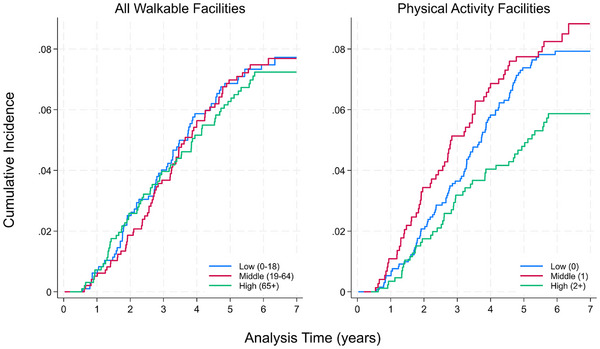
Cumulative incidence of mixed/vascular dementia by tertiles of neighborhood establishment measures (*N* = 2923). *Note*: Plots were truncated at 7 years given low sample size with follow‐up > 7 years (*n* = 159, 5%).

The cumulative incidence of mixed/vascular dementia over 8 years differed by tertiles of nearby physical activity facilities, but not all walkable facilities (Figure 1). Notably, although individuals with one nearby physical activity establishment (second tertile) appeared to have a higher cumulative incidence compared to those with zero facilities, these differences were not statistically significant (Table 3).

**TABLE 3 alz14387-tbl-0003:** Adjusted associations between neighborhood physical activity facilities within 1 km and incident mixed/vascular dementia (*N* = 2923).

	Model 1^a^		Model 2^b^		Model 3^c^		Model 4^d^		Model 5^e^	
	HR (95% CI)	*p* value	HR (95% CI)	*p* value	HR (95% CI)	*p* value	HR (95% CI)	*p* value	HR (95% CI)	*p* value
PA facilities (Ref: Low [0])										
Middle (1)	1.08 (0.76, 1.54)	0.654	1.11 (0.78, 1.59)	0.548	1.14 (0.78, 1.66)	0.504	1.16 (0.80, 1.69)	0.439	1.24 (0.82, 1.88)	0.311
High (≥ 2)	0.65 (0.45, 0.94)	0.024	0.61 (0.39, 0.96)	0.030	0.66 (0.42, 1.03)	0.067	0.70 (0.45, 1.10)	0.120	0.56 (0.35, 0.89)	0.015
nSES (Ref: Low)										
Middle							0.70 (0.50, 0.99)	0.041	0.69 (0.46, 1.03)	0.068
High							0.68 (0.47, 0.98)	0.039	0.76 (0.50, 1.17)	0.210

*Note*: Estimates are hazard ratios of risk of incident mixed/vascular dementia for higher tertiles compared to the lowest tertile of the neighborhood measure (PA facilities, nSES). nSES tertiles: low = –5.7, ‐1.6, middle = –1.6, 1.3, high = 1.3, 25.8).

Abbreviations: CHS, Cardiovascular Health Study; CI, confidence interval; HR, hazard ratio, PA, physical activity, nSES, neighborhood socioeconomic status index.

^a^ Model 1 was age adjusted.

^b^ Model 2 further adjusted for sex (man, woman), race (Black/African American, White), marital status (married, widowed, divorced/separated, never married), study site (Hagerstown, MD; Pittsburgh, PA; Sacramento, CA; Winston‐Salem, NC), residential mobility (any change in address from 1989–1993, yes/no), and urbanicity (tertiles of population density within 1 km).

^c^ Model 3 further adjusted for individual education (< high school, high school degree/General Educational Development degree, > high school), income (< $12k, [$12–25k), [$25–35k), ≥ $35k, missing), and lifetime occupation (professional, service, laborer, housewife, other).

^d^ Model 4 further adjusted for neighborhood socioeconomic status (nSES).

^e^ Model 5 excluded individuals in bottom 20th percentile of baseline physical activity (≤ 225 kcal/week).

Compared to having 0 physical activity facilities (lowest tertile), having ≥ 2 facilities (highest tertile) was associated with a 35% reduced hazard of incident mixed/vascular dementia in the age‐adjusted model (Table 3, Model 1, HR = 0.65, 95% CI: 0.45, 0.94). This association was attenuated after full covariate adjustment, including nSES (Model 3, HR = 0.70, 95% CI: 0.45, 1.10), but was strengthened after further excluding individuals with low physical activity (Model 4: HR = 0.56, 95% CI: 0.35, 0.89).

We found no significant associations between physical activity facilities and incident all‐cause dementia or AD only subtype (Table S1 in supporting information). Compared to the smaller buffer size (1 km), physical activity facilities measured within 5 km (i.e., driving distance) generally had weaker associations with mixed/vascular dementia (Table S2 in supporting information). We also found no significant associations between all walkable facilities within 1 km and any dementia outcome (Table S3 in supporting information). Finally, associations between physical activity facilities and incident dementia outcomes did not appear to differ by race (Tables S4 and S5 in supporting information).

## 4 DISCUSSION

We examined both aggregate nSES and specific neighborhood facility measures as potential environmental contributors to dementia risk in later life. We found that greater nSES was associated with a lower risk of incident mixed/vascular dementia. Additionally, dementia risk differed by number of physical activity facilities within 1 km of the home. Specifically, having ≥ 2 nearby physical activity facilities was associated with a reduced risk of incident mixed/vascular dementia, and this relationship was modestly attenuated after adjusting for individual and neighborhood SES. No neighborhood measures were associated with incident AD subtype after covariate adjustment. These findings emphasize the need to consider the neighborhood built environment in addition to individual‐level contributors to dementia risk in later life.[Table alz14387-tbl-0003]

Our finding that greater nSES was associated with reduced risk of incident mixed/vascular dementia parallels findings from prior studies.^12^, ^42^ For example, lower ADI scores (i.e., higher nSES) have been linked with lower risk of all‐cause dementia and dementia‐related death.^12^ This association may be driven by structural and functional brain changes, as lower ADI scores have further predicted reduced cortical thinning in ADRD‐related regions (e.g., entorhinal cortex) and higher cognitive functioning for older adults.^11^ In contrast, George et al. found that nSES did not predict dementia risk after accounting for individual SES in midlife adults.^14^ Our findings may have differed here because participants in our study were older and we also examined dementia subtypes, with mixed/vascular dementia appearing to have the strongest relationship with nSES. nSES likely operates through multiple behavioral mechanisms, including providing better quality amenities or encouraging specific norms around health behaviors.^19^, ^31^

Higher SES neighborhoods may also offer a greater number of businesses and services that enrich health. In the current study, higher nSES was associated with greater number of facilities within 1 km of home. We found no relationship between dementia and the number of all walkable facilities, potentially because this broad category also included facilities with a complex relationship with cognitive health. For example, this measure included fast‐food restaurants, which lack nutritional food options important for neurovascular health but also may encourage cognitively enriching social engagement.^43^ We did find that living in an area with ≥ 2 nearby physical activity facilities was associated with reduced risk of incident mixed/vascular dementia. These facilities included fitness, recreation, or multiuse facilities (e.g., YMCAs) that typically encourage higher levels of physical activity. Later‐life physical activity may protect against dementia by promoting cerebrovascular health, reducing neuroinflammation, and enhancing neurotrophic expression.^2^, ^3^, ^4^

Prior studies have found associations between a greater number of neighborhood physical activity facilities and cognitive performance in later life.^17^, ^19^, ^20^, ^21^ For example, Finlay et al. observed that higher tract‐level density of recreation facilities (e.g., gyms, golf course, tennis courts) was correlated with higher global cognitive performance for adults age ≥ 45.^17^ In a cohort of older adults in Chicago, living in neighborhoods with a greater number of community centers was also associated with slower cognitive declines.^21^ While we found no associations with total walkable facilities, Besser et al. further found that a greater density of walking destinations (e.g., post offices, banks, drug stores) was related to greater processing speed for a racially diverse cohort of older adults.^44^ Notably, these studies largely focused on cognitive performance measures. Here we demonstrated that neighborhood physical activity facilities are further associated with adjudicated mixed/vascular dementia, a clinical outcome characterized by severe cognitive and functional impairments.

Importantly, we found that the relationship between physical activity facilities and mixed/vascular dementia was only significant for facilities counted within 1 km (versus 5 km) of the home. There was also no observed benefit of having only one nearby facility. This may highlight the importance of both geographic accessibility and availability (i.e., enough quantity) of these resources to mitigate dementia risk in later life. For example, older adults in particular may be more likely to use a gym or recreation center if it is close to home and if there are enough facilities available to limit overcrowding.^45^, ^46^ Finally, excluding individuals with low baseline physical activity strengthened the association between physical activity facilities and incident mixed/vascular dementia. This suggests that increased availability of physical activity facilities may promote neurocognitive health primarily for those with sufficient mobility to access and use these resources.

Contrary to our hypothesis, we found no associations between any neighborhood measure and incident AD subtype after covariate adjustment. Because cases with only AD did not present with substantial vascular pathology (e.g., brain infarcts),^23^, ^24^ this may again suggest that these neighborhood resources mitigate dementia risk primarily through a vascular pathway. Supporting this, a prior CHS analysis found that the quantity of nearby physical activity facilities also predicted lower risk of incident cardiovascular disease.^25^ These facilities may promote vascular health through encouraging physical activity, and some studies have observed a stronger protective association of physical activity for vascular dementia compared to AD.^47^, ^48^, ^49^ Future studies could examine the neurocognitive benefits of neighborhood facilities that specifically encourage cognitive activity (e.g., library, arts/performance), which may be more strongly associated with AD.^47^, ^50^

### 4.1 Limitations and strengths

Our study was restricted to Black/African American and White participants from a specific period (1992–1999), which limits generalizability to the current population of US older adults. We also did not have access to residential histories of participants, making us unable to assess changes in address prior to study entry and account for the length of exposure to neighborhood resources across the life course. Yet, NETS establishment data were not available before the start of this study (pre‐1990),^32^ so an important future direction is to evaluate the cumulative impact of neighborhood facilities on dementia outcomes as these data continue to be collected in ongoing cohort studies. Changes in neighborhood built and socioeconomic characteristics also typically occur gradually over many years.^51^, ^52^ Therefore, studies with more longitudinal follow‐up could further examine whether changes in walkable facilities mediate the relationship between nSES and dementia.

We were unable to evaluate participants’ use of nearby facilities, but in supplemental analyses we excluded individuals with very low baseline activity who may have been less likely to use these resources. Wearable Global Positioning Systems (GPS)^53^ or mental mapping^54^ approaches offer promise for better exploring how engagement with the neighborhood environment may influence neurocognitive health in later life. Future studies could also quantify neighborhood resources using road network buffers.^55^, ^56^ Compared to the Euclidean (i.e., straight‐line) buffers used here, road network buffers may better account for the surrounding geographic context (natural barriers, street connectivity) important for accessing neighborhood resources.

Our study also had several strengths. CHS is a large, well‐characterized cohort of community‐dwelling older adults with dementia outcomes adjudicated from multiple data sources. Dementia was further classified by subtype, which was important given our findings for all‐cause dementia appeared to be driven primarily by mixed/vascular cases. It will be important to further replicate these findings in studies incorporating more recent biomarker measures (e.g., cerebrospinal fluid/plasma tau and amyloid) that may improve diagnostic accuracy.^57^ Our prospective study design also provided better support for the temporal precedence of neighborhood measures on incident dementia compared to prior cross‐sectional reports. Facility measures were generated using NETS, one of the most comprehensive data sources of US businesses.^32^ To our knowledge, we are among the first to use this extensive data source to examine links between facilities that specifically promote physical exercise and dementia risk. This expands upon prior work that has largely studied aggregate socioeconomic indices.

## 5 CONCLUSION

Growing evidence suggests that the neighborhood built environment may promote neurocognitive health in later life. Our findings suggest that the availability of nearby facilities that encourage physical exercise may be one specific structural mechanism that could mitigate risk of mixed/vascular dementia. Compared to individual‐level interventions, addressing upstream environmental factors has the potential to benefit the broader population and reduce structural disparities in neurocognitive health as we age. Future studies can build upon this work by examining the cumulative effect of neighborhood facilities across the life course on dementia risk in later life, as these measures continue to be collected in ongoing cohorts.

## CONFLICT OF INTEREST STATEMENT

None. Author disclosures are available in the supporting information.

## CONSENT STATEMENT

All human subjects provided informed consent.

## Supporting information



Supporting Information

Supporting Information

## References

[1] McKhann GM , Knopman DS , Chertkow H , et al. The diagnosis of dementia due to Alzheimer's disease: recommendations from the National Institute on Aging‐Alzheimer's Association workgroups on diagnostic guidelines for Alzheimer's disease. Alzheimers Dement. 2011;7(3):263‐269. doi:10.1016/j.jalz.2011.03.005 21514250 PMC3312024

[2] National Academies of Sciences, Engineering, and Medicine . Preventing Cognitive Decline and Dementia: A Way Forward. National Academies Press; 2017. 28650595

[3] Carlson MC , Varma VR . Activity and neurocognitive health in older adults. In: Waldstein SR , Elias MF , eds. Neuropsychology of Cardiovascular Disease. Psychology Press; 2015.

[4] Livingston G , Huntley J , Sommerlad A , et al. Dementia prevention, intervention, and care: 2020 report of the Lancet Commission. The Lancet. 2020;396(10248):413‐446. doi:10.1016/s0140-6736(20)30367-6 PMC7392084 32738937

[5] Link BG , Phelan J . Social conditions as fundamental causes of disease. J Health Soc Behav. 1995(Spec No):80‐94. 7560851

[6] Adkins‐Jackson PB , George KM , Besser LM , et al. The structural and social determinants of Alzheimer's disease related dementias. Alzheimers Dement. 2023;19:3171‐3185. doi:10.1002/alz.13027 37074203 PMC10599200

[7] Rosso AL , Flatt JD , Carlson MC , et al. Neighborhood socioeconomic status and cognitive function in late life. Am J Epidemiol. 2016;183(12):1088‐1097. doi:10.1093/aje/kwv337 27257114 PMC4908209

[8] Shih RA , Ghosh‐Dastidar B , Margolis KL , et al. Neighborhood socioeconomic status and cognitive function in women. Am J Public Health. 2011;101(9):1721‐1728. doi:10.2105/AJPH.2011.300169 21778482 PMC3154213

[9] Lamar M , Kershaw KN , Leurgans SE , et al. Neighborhood‐level social vulnerability and individual‐level cognitive and motor functioning over time in older non‐Latino Black and Latino adults. Front Hum Neurosci. 2023;17:1125906. doi:10.3389/fnhum.2023.1125906 37250695 PMC10213534

[10] Kind AJ , Buckingham WR . Making neighborhood‐disadvantage metrics accessible—the neighborhood atlas. N Engl J Med. 2018;378(26):2456. 29949490 10.1056/NEJMp1802313 PMC6051533

[11] Hunt JFV , Vogt NM , Jonaitis EM , et al. Association of neighborhood context, cognitive decline, and cortical change in an unimpaired cohort. Neurology. 2021;96(20):e2500‐e2512. doi:10.1212/WNL.0000000000011918 33853894 PMC8205478

[12] Becerril A , Pfoh ER , Hashmi AZ , et al. Racial, ethnic and neighborhood socioeconomic differences in incidence of dementia: a regional retrospective cohort study. J Am Geriatr Soc. 2023;71(8):2406‐2418. doi:10.1111/jgs.18322 36928611

[13] Meyer OL , Harrati A , Gavett BE , et al. Effects of early‐life environment and adulthood SES on cognitive change in a multiethnic cohort. J Int Neuropsychol Soc. 2023:1‐9. doi:10.1017/S135561772200087X Published online March 7 PMC10483016 36880230

[14] George KM , Lutsey PL , Kucharska‐Newton A , et al. Life‐course individual and neighborhood socioeconomic status and risk of dementia in the atherosclerosis risk in communities neurocognitive study. Am J Epidemiol. 2020;189(10):1134‐1142. doi:10.1093/aje/kwaa072 32383452 PMC7666419

[15] Chen X , Lee C , Huang H . Neighborhood built environment associated with cognition and dementia risk among older adults: a systematic literature review. Soc Sci Med. 2022;292:114560. doi:10.1016/j.socscimed.2021.114560 34776284

[16] Rundle AG , Chen Y , Quinn JW , et al. Development of a neighborhood walkability index for studying neighborhood physical activity contexts in communities across the U.S. over the past three decades. J Urban Health. 2019;96(4):583‐590. doi:10.1007/s11524-019-00370-4 31214976 PMC6677835

[17] Finlay J , Esposito M , Langa KM , Judd S , Clarke P . Cognability: an ecological theory of neighborhoods and cognitive aging. Soc Sci Med. 2022:115220. doi:10.1016/j.socscimed.2022.115220 Published online July 20 35926362 PMC9661364

[18] Hirsch JA , Moore KA , Cahill J , et al. Business data categorization and refinement for application in longitudinal neighborhood health research: a methodology. J Urban Health. 2021;98(2):271‐284. doi:10.1007/s11524-020-00482-2 33005987 PMC8079597

[19] Clarke PJ , Ailshire JA , House JS , et al. Cognitive function in the community setting: the neighbourhood as a source of ‘cognitive reserve’?. J Epidemiol Community Health. 2012;66(8):730‐736. doi:10.1136/jech.2010.128116 21515547 PMC3387518

[20] Yang HW , Wu YH , Lin MC , et al. Association between neighborhood availability of physical activity facilities and cognitive performance in older adults. Prev Med. 2023;175:107669. doi:10.1016/j.ypmed.2023.107669 37595898

[21] Clarke PJ , Weuve J , Barnes L , Evans DA , Mendes de Leon CF . Cognitive decline and the neighborhood environment. Ann Epidemiol. 2015;25(11):849‐854. doi:10.1016/j.annepidem.2015.07.001 26253697 PMC4609590

[22] Luo Y , Zhang L , Pan X . Neighborhood environments and cognitive decline among middle‐aged and older people in China. J Gerontol Ser B. 2019;74(7):e60‐e71. doi:10.1093/geronb/gbz016 30726959

[23] Lopez OL , Kuller LH , Fitzpatrick A , Ives D , Becker JT , Beauchamp N . Evaluation of dementia in the cardiovascular health cognition study. Neuroepidemiology. 2003;22(1):1‐12. doi:10.1159/000067110 12566948

[24] Fitzpatrick AL , Kuller LH , Ives DG , et al. Incidence and prevalence of dementia in the cardiovascular health study. J Am Geriatr Soc. 2004;52(2):195‐204. doi:10.1111/j.1532-5415.2004.52058.x 14728627

[25] Garg PK , Platt JM , Hirsch JA , et al. Association of neighborhood physical activity opportunities with incident cardiovascular disease in the Cardiovascular Health Study. Health Place. 2021;70:102596. doi:10.1016/j.healthplace.2021.102596 34091144 PMC8328953

[26] Drexel University Urban Health Collaborative . Retail environment and cardiovascular disease (RECVD). Published online 2023. https://drexel.edu/uhc/research/projects/retail‐environment‐cardiovascular‐disease/

[27] Godina SL , Rosso AL , Hirsch JA , et al. Neighborhood greenspace and cognition: the cardiovascular health study. Health Place. 2023;79:102960. doi:10.1016/j.healthplace.2022.102960 36603455 PMC9928891

[28] Lovasi GS , Neckerman KM , Quinn JW , Weiss CC , Rundle A . Effect of individual or neighborhood disadvantage on the association between neighborhood walkability and body mass index. Am J Public Health. 2009;99(2):279‐284. doi:10.2105/AJPH.2008.138230 19059849 PMC2622783

[29] Logan JR , Xu Z , Stults BJ . Interpolating U.S. decennial census tract data from as early as 1970 to 2010: a longitudinal tract database. Prof Geogr. 2014;66(3):412‐420. doi:10.1080/00330124.2014.905156 25140068 PMC4134912

[30] Diez Roux AV . Neighbourhood environments and mortality in an elderly cohort: results from the cardiovascular health study. J Epidemiol Community Health. 2004;58(11):917‐923. doi:10.1136/jech.2003.019596 15483307 PMC1732601

[31] Diez Roux AV , Kiefe CI , Jacobs DR , et al. Area characteristics and individual‐level socioeconomic position indicators in three population‐based epidemiologic studies. Ann Epidemiol. 2001;11(6):395‐405. doi:10.1016/S1047-2797(01)00221-6 11454499

[32] Kaufman TK , Sheehan DM , Rundle A , et al. Measuring health‐relevant businesses over 21 years: refining the National Establishment Time‐Series (NETS), a dynamic longitudinal data set. BMC Res Notes. 2015;8(1):507. doi:10.1186/s13104-015-1482-4 26420471 PMC4588464

[33] American Psychiatric Association . Diagnostic and Statistical Manual of Mental Disorders : DSM‐IV‐TR. 4th ed. American Psychiatric Association; 2000.

[34] McKhann G , Drachman D , Folstein M , Katzman R , Price D , Stadlan EM . Clinical diagnosis of Alzheimer's disease Report of the NINCDS‐ADRDA Work Group* under the auspices of department of health and human services task force on Alzheimer's Disease. Neurology. 1984;34(7):939‐939. doi:10.1212/WNL.34.7.939 6610841

[35] Romain G , Tatemichi T , Erkinjuntti T , et al. Vascular dementia: diagnostic criteria for research studies. Report of the NINDS‐AIREN International Workshop. Neurology. 1993;43(2):250‐260. 8094895 10.1212/wnl.43.2.250

[36] Chui HC , Victoroff JI , Margolin D , Jagust W , Shankle R , Katzman R . Criteria for the diagnosis of ischemic vascular dementia proposed by the State of California Alzheimer's Disease Diagnostic and Treatment Centers. Neurology. 1992;42(3):473‐473. doi:10.1212/WNL.42.3.473 1549205

[37] Podewils LJ , Guallar E , Kuller LH , et al. Physical activity, APOE genotype, and dementia risk: findings from the Cardiovascular Health Cognition Study. Am J Epidemiol. 2005;161(7):639‐651. doi:10.1093/aje/kwi092 15781953

[38] Taylor HL , Jacobs DR , Schucker B , Knudsen J , Leon AS , Debacker G . A questionnaire for the assessment of leisure time physical activities. J Chronic Dis. 1978;31(12):741‐755. doi:10.1016/0021-9681(78)90058-9 748370

[39] Austin PC , Lee DS , Fine JP . Introduction to the analysis of survival data in the presence of competing risks. Circulation. 2016;133(6):601‐609. doi:10.1161/CIRCULATIONAHA.115.017719 26858290 PMC4741409

[40] Fried LP , Tangen CM , Walston J , et al. Frailty in older adults: evidence for a phenotype. J Gerontol Ser A. 2001;56(3):M146‐M157. doi:10.1093/gerona/56.3.M146 11253156

[41] StataCorp . Stata Statistical Software: Release 18. Published online 2023.

[42] Pase MP , Rowsthorn E , Cavuoto MG , et al. Association of neighborhood‐level socioeconomic measures with cognition and dementia risk in Australian Adults. JAMA Netw Open. 2022;5(3):e224071‐e224071. doi:10.1001/jamanetworkopen.2022.4071 35333361 PMC8956972

[43] Finlay J , Esposito M , Tang S , et al. Fast‐food for thought: retail food environments as resources for cognitive health and wellbeing among aging Americans?. Health Place. 2020;64:102379. doi:10.1016/j.healthplace.2020.102379 32838895 PMC7480653

[44] Besser LM , Chang LC , Hirsch JA , et al. Longitudinal associations between the neighborhood built environment and cognition in US older adults: the multi‐ethnic study of atherosclerosis. Int J Environ Res Public Health. 2021;18(15):7973. doi:10.3390/ijerph18157973 34360264 PMC8345405

[45] Lübcke A , Martin C , Hellström K . Older adults’ perceptions of exercising in a senior gym. Act Adapt Aging. 2012;36(2):131‐146. doi:10.1080/01924788.2012.673157

[46] Cohen‐Mansfield J , Marx MS , Guralnik JM . Motivators and barriers to exercise in an older community‐dwelling population. J Aging Phys Act. 2003;11(2):242‐253. doi:10.1123/japa.11.2.242

[47] Najar J , Östling S , Gudmundsson P , et al. Cognitive and physical activity and dementia: a 44‐year longitudinal population study of women. Neurology. 2019. doi:10.1212/WNL.0000000000007021 Published online February 20 PMC6511097 30787164

[48] Iso‐Markku P , Kujala UM , Knittle K , Polet J , Vuoksimaa E , Waller K . Physical activity as a protective factor for dementia and Alzheimer's disease: systematic review, meta‐analysis and quality assessment of cohort and case–control studies. Br J Sports Med. 2022;56(12):701‐709. doi:10.1136/bjsports-2021-104981 35301183 PMC9163715

[49] Hansson O , Svensson M , Gustavsson AM , et al. Midlife physical activity is associated with lower incidence of vascular dementia but not Alzheimer's disease. Alzheimers Res Ther. 2019;11(1):87. doi:10.1186/s13195-019-0538-4 31630687 PMC6802179

[50] Wilson RS , Bennett DA . Cognitive activity and risk of Alzheimer's disease. Curr Dir Psychol Sci. 2003;12(3):87‐91. doi:10.1111/1467-8721.01236

[51] Hirsch JA , Grengs J , Schulz A , et al. How much are built environments changing, and where?: patterns of change by neighborhood sociodemographic characteristics across seven U.S. metropolitan areas. Soc Sci Med. 2016;169:97‐105. doi:10.1016/j.socscimed.2016.09.032 27701020 PMC5075249

[52] Delmelle EC . Differentiating pathways of neighborhood change in 50 U.S. metropolitan areas. Environ Plan Econ Space. 2017;49(10):2402‐2424. doi:10.1177/0308518X17722564

[53] Crane BM , Moored KD , Rosso AL , Carlson MC . Using GPS technologies to examine community mobility in older adults. J Gerontol Ser A. doi:10.1093/gerona/glac185 Published online September 8, 2022:glac185 PMC10172976 36073676

[54] Bödeker M . Walking and walkability in pre‐set and self‐defined neighborhoods: a mental mapping study in older adults. Int J Environ Res Public Health. 2018;15(7):1363. doi:10.3390/ijerph15071363 29958469 PMC6068775

[55] Frank LD , Appleyard BS , Ulmer JM , Chapman JE , Fox EH . Comparing walkability methods: creation of street smart walk score and efficacy of a code‐based 3D walkability index. J Transp Health. 2021;21:101005. doi:10.1016/j.jth.2020.101005

[56] James P , Berrigan D , Hart JE , et al. Effects of buffer size and shape on associations between the built environment and energy balance. Health Place. 2014;27:162‐170. doi:10.1016/j.healthplace.2014.02.003 24607875 PMC4028172

[57] Simrén J , Leuzy A , Karikari TK , et al. The diagnostic and prognostic capabilities of plasma biomarkers in Alzheimer's disease. Alzheimers Dement. 2021;17(7):1145‐1156. doi:10.1002/alz.12283 33491853 PMC8359457

