# Supplementary material for: Neighborhood physical activity facilities predict risk of incident mixed and vascular dementia: The Cardiovascular Health Cognition Study
Source: Alzheimers Dement. 2024 Nov 19;21(1):e14387. doi: 10.1002/alz.14387 (PMC11772720; doi:10.1002/alz.14387)
Supplement: Supplementary file 1 — Supporting Information [file ALZ-21-e14387-s002.docx]

**Neighborhood physical activity facilities predict risk of incident mixed and vascular dementia: The Cardiovascular Health Cognition Study**

**Supplemental Appendix**

Table S1: Adjusted Associations between Neighborhood Physical Activity Facilities within 1-km and Incident All-Cause Dementia and Alzheimer’s Disease Only Subtype

Table S2: Adjusted Associations between Neighborhood Physical Activity Facilities within 5-km and Incident Dementia Outcomes

Table S3: Adjusted Associations between Neighborhood Walkable Facilities within 1-km and Incident Dementia Outcomes

Table S4. Race Stratified Adjusted Associations between Neighborhood Physical Activity Facilities within 1-km and Incident Mixed/Vascular Dementia

Table S5. Race Stratified Adjusted Associations between Neighborhood Physical Activity Facilities within 1-km and Incident Alzheimer’s Disease Only

**Supplemental Results**

**Race Differences in Associations between** **Neighborhood Facilities and Incident Dementia**

We did not find conclusive evidence for race differences in the association between number of PA facilities within 1-km and risk of incident mixed/vascular dementia. The association for the highest tertile (≥2 PA facilities) among white individuals without low activity trended towards statistical significance (HR=.63, 95%CI: .38, 1.05, p=.075, Table S4), but effect sizes for Black participants were larger, despite not being statistically significant (HR’s=.43-.52, p’s>.10, Table S4). These findings warrant caution given that there was lower statistical power to detect differences in the smaller sample of Black/African American participants (n=414) compared to the larger white sample in this study (n=2509).” There were no associations between PA facilities and incident Alzheimer’s disease regardless of race (Table S5).

**Table S1:** **Adjusted Associations between Neighborhood Physical Activity Facilities within 1-km and Incident All-Cause Dementia and Alzheimer’s Disease Only Subtype**

|  | Model 1 (Age) | Model 2 (+Gender, Race, Study Site) | Model 3 (+Individual Education, Income, Occupation) | Model 4 (+nSES) | Model 5 (excluding those in bottom 20% of PA) |
| --- | --- | --- | --- | --- | --- |
|  | HR (95% CI) | HR (95% CI) | HR (95% CI) | HR (95% CI) | HR (95% CI) |
| **All-Cause Dementia** |  |  |  |  |  |
|  |  |  |  |  |  |
| PA Facilities  (Ref: Low (0)) |  |  |  |  |  |
| Middle (1) | 1.00 (0.77, 1.30) | 1.06 (0.82, 1.38) | 1.12 (0.86, 1.47) | 1.14 (0.87, 1.49) | 1.16 (0.86, 1.55) |
| High (≥2) | 0.80 (0.62, 1.04) | 0.82 (0.62, 1.10) | 0.89 (0.68, 1.17) | 0.92 (0.70, 1.22) | 0.82 (0.60, 1.13) |
|  |  |  |  |  |  |
| nSES  (Ref: Low (-5.7 - -1.6)) |  |  |  |  |  |
| Middle (-1.6 - 1.3) |  |  |  | 0.85 (0.66, 1.08) | 0.88 (0.66, 1.17) |
| High (1.3 - 25.8) |  |  |  | 0.83 (0.64, 1.07) | 0.84 (0.62, 1.13) |
| **Alzheimer’s Only Subtype** |  |  |  |  |  |
|  |  |  |  |  |  |
| PA Facilities  (Ref: Low (0)) |  |  |  |  |  |
| Middle (1) | 0.97 (0.69, 1.35) | 1.03 (0.73, 1.47) | 1.11 (0.78, 1.59) | 1.12 (0.78, 1.60) | 1.06 (0.71, 1.59) |
| High (≥2) | 0.89 (0.65, 1.23) | 0.98 (0.67, 1.44) | 1.05 (0.72, 1.54) | 1.07 (0.73, 1.58) | 1.03 (0.66, 1.61) |
|  |  |  |  |  |  |
| nSES  (Ref: Low (-5.7 - -1.6)) |  |  |  |  |  |
| Middle (-1.6 - 1.3) |  |  |  | 0.93 (0.66, 1.31) | 1.01 (0.68, 1.50) |
| High (1.3 - 25.8) |  |  |  | 0.91 (0.62, 1.33) | 0.85 (0.54, 1.34) |

Note. Estimates are hazard ratios of risk of incident dementia for higher tertiles compared to the lowest tertile for each neighborhood measure (PA facilities, nSES). Model 1 was age-adjusted. Model 2 further adjusted for gender (man, woman), race (Black/African-American, White), study site (Hagerstown, MD; Pittsburgh, PA; Sacramento, CA; Winston-Salem, NC), marital status (married, widowed, divorced/separated, never married), study site (Hagerstown, MD; Pittsburgh, PA; Sacramento, CA; Winston-Salem, NC), residential mobility (any change in address from 1989-1993, yes/no), urbanicity (tertiles of population density within 1-km). Model 3 further adjusted for individual education (<high school, high school degree/GED, >high school), income (<$12k, [$12-25k), [$25-35k), ≥$35k, missing), and lifetime occupation (professional, service, laborer, housewife, other). Model 5 excluded individuals in bottom 20^th^ percentile of baseline physical activity (≤225 kcal/week).

PA = physical activity, HR = hazard ratio, nSES = neighborhood socioeconomic status index

**Table S2:** **Adjusted Associations between Neighborhood Physical Activity Facilities within 5-km and Incident Dementia Outcomes**

|  | Model 1 (Age) | Model 2 (+Gender, Race, Study Site) | Model 3 (+Individual Education, Income, Occupation) | Model 4 (+nSES) | Model 5 (excluding those in bottom 20% of PA) |
| --- | --- | --- | --- | --- | --- |
|  | HR (95% CI) | HR (95% CI) | HR (95% CI) | HR (95% CI) | HR (95% CI) |
| **All-Cause Dementia** |  |  |  |  |  |
|  |  |  |  |  |  |
| PA Facilities  (Ref: Low (0-14)) |  |  |  |  |  |
| Middle (15-29) | 0.97 (0.76, 1.24) | 1.00 (0.77, 1.29) | 1.02 (0.79, 1.32) | 1.00 (0.77, 1.29) | 0.97 (0.72, 1.30) |
| High (≥30) | 0.78 (0.60, 1.02) | 0.77 (0.52, 1.14) | 0.85 (0.58, 1.27) | 0.86 (0.58, 1.27) | 0.67 (0.42, 1.07) |
|  |  |  |  |  |  |
| nSES  (Ref: Low (-11.7 - -1.3)) |  |  |  |  |  |
| Middle (-1.3 - 0.99) |  |  |  | 0.86 (0.66, 1.13) | 0.90 (0.67, 1.22) |
| High (1.00 - 22.8) |  |  |  | 1.05 (0.83, 1.33) | 0.85 (0.63, 1.15) |
| **Alzheimer’s Only Subtype** |  |  |  |  |  |
|  |  |  |  |  |  |
| PA Facilities  (Ref: Low (0-14)) |  |  |  |  |  |
| Middle (15-29) | 0.92 (0.68, 1.27) | 0.88 (0.60, 1.31) | 0.89 (0.59, 1.33) | 0.89 (0.58, 1.35) | 0.91 (0.57, 1.44) |
| High (≥30) | 0.78 (0.57, 1.06) | 0.78 (0.45, 1.36) | 0.87 (0.50, 1.51) | 0.83 (0.47, 1.47) | 0.80 (0.41, 1.55) |
|  |  |  |  |  |  |
| nSES  (Ref: Low (-11.7 - -1.3)) |  |  |  |  |  |
| Middle (-1.3 - 0.99) |  |  |  | 1.18 (0.82, 1.70) | 1.02 (0.68, 1.53) |
| High (1.00 - 22.8) |  |  |  | 1.16 (0.81, 1.67) | 0.87 (0.55, 1.38) |
| **Mixed/Vascular Subtype** |  |  |  |  |  |
|  |  |  |  |  |  |
| PA Facilities  (Ref: Low (0-14)) |  |  |  |  |  |
| Middle (15-29) | 1.02 (0.70, 1.49) | 1.05 (0.70, 1.57) | 1.09 (0.73, 1.63) | 1.04 (0.71, 1.52) | 0.96 (0.60, 1.54) |
| High (≥30) | 0.74 (0.50, 1.09) | 0.66 (0.36, 1.20) | 0.73 (0.40, 1.34) | 0.75 (0.41, 1.36) | 0.51 (0.25, 1.04) |
|  |  |  |  |  |  |
| nSES  (Ref: Low (-11.7 - -1.3)) |  |  |  |  |  |
| Middle (-1.3 - 0.99) |  |  |  | 0.64 (0.43, 0.95)* | 0.72 (0.47, 1.09) |
| High (1.00 - 22.8) |  |  |  | 1.01 (0.70, 1.46) | 0.76 (0.50, 1.17) |

Note. Estimates are hazard ratios of risk of incident dementia for higher tertiles compared to the lowest tertile for each neighborhood measure (PA facilities, nSES). Model 1 was age-adjusted. Model 2 further adjusted for gender (man, woman), race (Black/African-American, White), study site (Hagerstown, MD; Pittsburgh, PA; Sacramento, CA; Winston-Salem, NC), marital status (married, widowed, divorced/separated, never married), study site (Hagerstown, MD; Pittsburgh, PA; Sacramento, CA; Winston-Salem, NC), residential mobility (any change in address from 1989-1993, yes/no), urbanicity (tertiles of population density within 1-km). Model 3 further adjusted for individual education (<high school, high school degree/GED, >high school), income (<$12k, [$12-25k), [$25-35k), ≥$35k, missing), and lifetime occupation (professional, service, laborer, housewife, other). Model 5 excluded individuals in bottom 20^th^ percentile of baseline physical activity (≤225 kcal/week).

PA = physical activity, HR = hazard ratio, nSES = neighborhood socioeconomic status index, CHS = Cardiovascular Health Study

*p<.05.

**Table S3: Adjusted Associations between Neighborhood Walkable Facilities within 1-km and Incident Dementia Outcomes**

|  | Model 1 (Age) | Model 2 (+Gender, Race, Study Site) | Model 3 (+Individual Education, Income, Occupation) | Model 4 (+nSES) | Model 5 (excluding those in bottom 20% of PA) |
| --- | --- | --- | --- | --- | --- |
|  | HR (95% CI) | HR (95% CI) | HR (95% CI) | HR (95% CI) | HR (95% CI) |
| **All-Cause Dementia** |  |  |  |  |  |
|  |  |  |  |  |  |
| Walkable Facilities  (Ref: Low (0-18)) |  |  |  |  |  |
| Middle (19-64) | 1.02 (0.78, 1.34) | 1.02 (0.76, 1.39) | 1.07 (0.80, 1.43) | 1.06 (0.79, 1.42) | 1.11 (0.79, 1.56) |
| High (≥65) | 0.94 (0.71, 1.23) | 1.02 (0.71, 1.46) | 1.05 (0.74, 1.49) | 1.07 (0.75, 1.52) | 1.04 (0.69, 1.58) |
|  |  |  |  |  |  |
| nSES  (Ref: Low (-5.7 - -1.6)) |  |  |  |  |  |
| Middle (-1.6 - 1.3) |  |  |  | 0.85 (0.66, 1.08) | 0.88 (0.66, 1.17) |
| High (1.3 - 25.8) |  |  |  | 0.81 (0.62, 1.04) | 0.81 (0.60, 1.08) |
| **Alzheimer’s Only Subtype** |  |  |  |  |  |
|  |  |  |  |  |  |
| Walkable Facilities  (Ref: Low (0-18)) |  |  |  |  |  |
| Middle (19-64) | 1.15 (0.83, 1.60) | 1.15 (0.77, 1.72) | 1.18 (0.81, 1.73) | 1.18 (0.80, 1.73) | 1.17 (0.74, 1.87) |
| High (≥65) | 1.03 (0.76, 1.41) | 1.17 (0.74, 1.84) | 1.19 (0.76, 1.88) | 1.20 (0.76, 1.91) | 1.29 (0.75, 2.22) |
|  |  |  |  |  |  |
| nSES  (Ref: Low (-5.7 - -1.6)) |  |  |  |  |  |
| Middle (-1.6 - 1.3) |  |  |  | 0.94 (0.67, 1.32) | 1.02 (0.69, 1.52) |
| High (1.3 - 25.8) |  |  |  | 0.91 (0.62, 1.34) | 0.84 (0.54, 1.32) |
| **Mixed/Vascular Subtype** |  |  |  |  |  |
|  |  |  |  |  |  |
| Walkable Facilities  (Ref: Low (0-18)) |  |  |  |  |  |
| Middle (19-64) | 0.93 (0.64, 1.36) | 0.94 (0.59, 1.48) | 0.99 (0.62, 1.57) | 0.97 (0.61, 1.53) | 1.05 (0.64, 1.71) |
| High (≥65) | 0.84 (0.57, 1.25) | 0.88 (0.50, 1.57) | 0.92 (0.52, 1.62) | 0.95 (0.54, 1.67) | 0.81 (0.43, 1.55) |
|  |  |  |  |  |  |
| nSES  (Ref: Low (-5.7 - -1.6)) |  |  |  |  |  |
| Middle (-1.6 - 1.3) |  |  |  | 0.69 (0.49, 0.97)* | 0.68 (0.45, 1.02) |
| High (1.3 - 25.8) |  |  |  | 0.63 (0.44, 0.91)* | 0.71 (0.46, 1.10) |

Note. Estimates are hazard ratios of risk of incident dementia for higher tertiles compared to the lowest tertile for each neighborhood measure (walkable facilities, nSES). Model 1 was age-adjusted. Model 2 further adjusted for gender (man, woman), race (Black/African-American, White), study site (Hagerstown, MD; Pittsburgh, PA; Sacramento, CA; Winston-Salem, NC), marital status (married, widowed, divorced/separated, never married), study site (Hagerstown, MD; Pittsburgh, PA; Sacramento, CA; Winston-Salem, NC), residential mobility (any change in address from 1989-1993, yes/no), urbanicity (tertiles of population density within 1-km). Model 3 further adjusted for individual education (<high school, high school degree/GED, >high school), income (<$12k, [$12-25k), [$25-35k), ≥$35k, missing), and lifetime occupation (professional, service, laborer, housewife, other). Model 5 excluded individuals in bottom 20^th^ percentile of baseline physical activity (≤225 kcal/week).

PA = physical activity, HR = hazard ratio, nSES = neighborhood socioeconomic status index, CHS = Cardiovascular Health Study

*p<.05.

**Table S4. Race Stratified Adjusted Associations between Neighborhood Physical Activity Facilities within 1-km and Incident Mixed/Vascular Dementia**

|  | **White** |  |  |  |
| --- | --- | --- | --- | --- |
|  | Overall Sample (n=2509) |  | Excluding Low PA (n=2046) |  |
|  | HR (95% CI) | P-value | HR (95% CI) | P-value |
| PA Facilities (Ref: Low (0)) |  |  |  |  |
| Middle (1) | 1.28 (0.85, 1.94) | 0.237 | 1.35 (0.85, 2.14) | 0.205 |
| High (≥2) | 0.80 (0.50, 1.27) | 0.335 | 0.63 (0.38, 1.05) | 0.075 |
|  |  |  |  |  |
| nSES (ref: Low (-5.1 - -1.2)) |  |  |  |  |
| Middle (-1.2 - 1.8) | 1.01 (0.67, 1.54) | 0.956 | 1.06 (0.67, 1.70) | 0.795 |
| High (1.8 - 25.8) | 0.69 (0.44, 1.06) | 0.090 | 0.73 (0.44, 1.20) | 0.212 |
|  |  |  |  |  |
|  | **Black/African-American** |  |  |  |
|  | Overall Sample (n=414) |  | Excluding Low PA (n=292) |  |
|  | HR (95% CI) | P-value | HR (95% CI) | P-value |
| PA Facilities (Ref: Low (0)) |  |  |  |  |
| Middle (1) | 0.72 (0.21, 2.50) | 0.601 | 1.40 (0.34, 5.73) | 0.639 |
| High (≥2) | 0.43 (0.14, 1.27) | 0.125 | 0.52 (0.15, 1.86) | 0.314 |
|  |  |  |  |  |
| nSES (ref: Low (-5.7 - -4.1)) |  |  |  |  |
| Middle (-4.0 - -1.9) | 0.79 (0.23, 2.80) | 0.719 | 1.21 (0.29, 5.15) | 0.796 |
| High (-1.9 - 7.8) | 0.81 (0.26, 2.56) | 0.726 | 0.85 (0.28, 2.57) | 0.773 |

Note. Estimates are hazard ratios of risk of incident mixed/vascular dementia for higher tertiles compared to the lowest tertile of the neighborhood measure (PA facilities, nSES).

Models were adjusted for age, gender (man, woman), marital status (married, widowed, divorced/separated, never married), study site (Hagerstown, MD; Pittsburgh, PA; Sacramento, CA; Winston-Salem, NC), residential mobility (any change in address from 1989-1993, yes/no), urbanicity (tertiles of population density within 1-km), individual education (<high school, high school degree/GED, >high school), income (<$12k, [$12-25k), [$25-35k), ≥$35k, missing), lifetime occupation (professional, service, laborer, housewife, other), and neighborhood socioeconomic status tertiles (nSES).

PA = physical activity, HR = hazard ratio, nSES = neighborhood socioeconomic status index, CHS = Cardiovascular Health Study

**Table S5. Race Stratified Adjusted Associations between Neighborhood Physical Activity Facilities within 1-km and Incident Alzheimer’s Disease Only**

|  | **White** |  |  |  |
| --- | --- | --- | --- | --- |
|  | Overall Sample (n=2509) |  | Excluding Low PA (n=2046) |  |
|  | HR (95% CI) | P-value | HR (95% CI) | P-value |
| PA Facilities (Ref: Low (0)) |  |  |  |  |
| Middle (1) | 1.08 (0.73, 1.60) | 0.699 | 1.12 (0.73, 1.72) | 0.616 |
| High (≥2) | 1.03 (0.68, 1.56) | 0.896 | 1.05 (0.64, 1.71) | 0.858 |
|  |  |  |  |  |
| nSES (ref: Low (-5.1 - -1.2)) |  |  |  |  |
| Middle (-1.2 - 1.8) | 0.81 (0.56, 1.17) | 0.258 | 0.86 (0.57, 1.30) | 0.477 |
| High (1.8 - 25.8) | 0.88 (0.60, 1.31) | 0.535 | 0.87 (0.55, 1.39) | 0.569 |
|  |  |  |  |  |
|  | **Black/African-American** |  |  |  |
|  | Overall Sample (n=414) |  | Excluding Low PA (n=292) |  |
|  | HR (95% CI) | P-value | HR (95% CI) | P-value |
| PA Facilities (Ref: Low (0)) |  |  |  |  |
| Middle (1) | 1.19 (0.34, 4.15) | 0.783 | 0.64 (0.13, 3.30) | 0.598 |
| High (≥2) | 1.20 (0.42, 3.41) | 0.739 | 1.35 (0.40, 4.52) | 0.626 |
|  |  |  |  |  |
| nSES (ref: Low (-5.7 - -4.1)) |  |  |  |  |
| Middle (-4.0 - -1.9) | 1.45 (0.58, 3.63) | 0.430 | 1.83 (0.63, 5.26) | 0.265 |
| High (-1.9 - 7.8) | 1.63 (0.48, 5.52) | 0.430 | 1.73 (0.39, 7.60) | 0.467 |

Note. Estimates are hazard ratios of risk of incident Alzheimer’s Disease for higher tertiles compared to the lowest tertile of the neighborhood measure (PA facilities, nSES).

Models were adjusted for age, gender (man, woman), marital status (married, widowed, divorced/separated, never married), study site (Hagerstown, MD; Pittsburgh, PA; Sacramento, CA; Winston-Salem, NC), residential mobility (any change in address from 1989-1993, yes/no), urbanicity (tertiles of population density within 1-km), individual education (<high school, high school degree/GED, >high school), income (<$12k, [$12-25k), [$25-35k), ≥$35k, missing), lifetime occupation (professional, service, laborer, housewife, other), and neighborhood socioeconomic status tertiles (nSES).

PA = physical activity, HR = hazard ratio, nSES = neighborhood socioeconomic status index, CHS = Cardiovascular Health Study
